# On-Chip Microplasmas for the Detection of Radioactive Cesium Contamination in Seawater

**DOI:** 10.3390/mi8090259

**Published:** 2017-08-23

**Authors:** Joshua B. Joffrion, Darrian Mills, William Clower, Chester G. Wilson

**Affiliations:** Institute for Micromanufacting, Louisiana Tech University, Ruston, LA 71272, USA; darrian.c.mills@gmail.com (D.M.); wclower@latech.edu (W.C.); chester@latech.edu (C.G.W.)

**Keywords:** microplasma device, plasma spectroscopy, cesium detection

## Abstract

On-chip microplasmas have previously been used in designing a compact and portable device for identifying pollutants in a water sample. By exciting a liquid sample with a high energy microdischarge and recording the spectral wavelengths emitted, the individual elements in the liquid are distinguishable. In particular, this study focuses on cesium, a contaminant from nuclear incidents such as the collapse of the nuclear power plant in Fukushima, Japan. This article shows that not only can the presence of cesium be clearly determined at concentrations as low as 10 ppb, but the relative concentration contained in the sample can be determined through the discharges’ relative spectral intensity.

## 1. Introduction

In March 2011, a 9.0 magnitude earthquake and the resulting 15-m tsunami caused the collapse of the Fukushima I Nuclear Power Plant in Fukushima, Japan, leading to a radiological incident requiring monitoring. Radioactive cesium, a waste product of nuclear power, demands concern as it is water-soluble and capable of affecting plants, animals, and water that we consume [1]. Soon after the incident, samples collected approximately 330 m from the Fukushima Daiichi Nuclear Power Station’s discharge canal contained 32,000 becquerels per liter (9.95 ppb) of cesium-137 and 31,000 becquerels per liter (0.70 ppb) of cesium-134 [2] (summing to 10.65 ppb of cesium). Cesium-137 has a half-life of about thirty years, and thus poses a long-term threat. The United States Environmental Protection Agency has established a Maximum Contaminant Level of cesium-137 in drinking water to be 200 picocuries per liter [3]. This equates to 7400 becquerels per cubic meter. Considering a kilogram of cesium-137 has an activity of 3.215 terabecquerel, this translates to 0.023 ppb. Cesium-133, the only stable isotope of cesium, occurs naturally in seawater at an average concentration ranging from 0.03 to 0.05 ppb [4].

Current methods of cesium detection generally require sample collecting and testing at off-site facilities [5]. However, some alternative methods do exist. Researchers at the Tokyo University of Science have developed an optode membrane that provides a visual response to the presence of cesium in water in the form of color change. Their optode membrane is fabricated using nanoparticles [6]. A group of researchers from the University of Rhode Island use fluorescence quenching for cesium detection. With the inclusion of a squaraine dye into partially aqueous solutions containing cesium metal ions, a reduction in fluorescence intensity can be observed. It is hypothesized that this is caused by a disruption of the donor–acceptor–donor architecture [7].

Our method of cesium detection employs microplasmas for on-site testing and monitoring of cesium contamination after a radiological incident. Microplasmas have earned a wide range of applications: from a nanotorch capable of generating very localized microplasma for new methods of controlled direct-write micro and nanofabrication [8] to electrohydrodynamic thrusters being developed that utilize corona discharges as a propulsion mechanism for flying insect-scale robots [9]. There has been no work found on the detection of cesium via plasma spectroscopy. However, work has already been done applying plasma spectroscopic techniques to on-site monitoring of the potability of water sources [10,11]. This paper reports on an extension of this idea into the detection of cesium in water samples. By exciting a liquid sample with a high energy microdischarge and recording the spectral wavelengths emitted, the individual elements in the liquid are distinguishable. Through the matching of multiple peaks to known cesium spectra, the presence of cesium in a water sample can be determined with certainty.

## 2. Device Design

**Figure 1:**
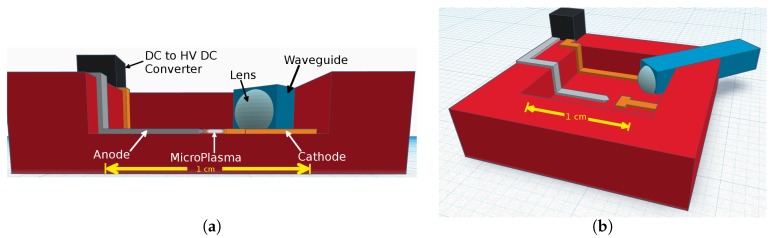
(**a**) Cross-section view of the device and its components. (**b**) 3D computer-aided design (CAD) model of the theoretical device.

The designed device is shown in Figure 1. Key components include a direct current (DC) to high voltage direct current (HV DC) converter, waveguide, lens, anode, and cathode. The lens ensures that the electromagnetic radiation emitted from the excited atoms enters the waveguide at an angle appropriate for total internal reflection to occur. The waveguide then ensures that a high percentage of the characteristic electromagnetic waves will be available for analysis. A DC to HV DC converter is used to amplify a small voltage supplied to the device to an output of several kilovolts [12]. Optical data is collected via an Ocean Optics HR2000CG-UV-NIR High-Resolution Spectrometer connected to a personal computer (PC).

The device is fabricated similarly to the device discussed by Sweeney et al. [11]. A micro-milled Teflon injection mold was used to batch fabricate alumina (Al${}_{2}$O${}_{3}$) ceramic substrate. Reservoirs for water samples with dimensions of 12 mm^2^ by 2 mm were etched into the ceramic substrate via sandblasting. The cathode and anode were patterned to a Cr film by spinning on S1813 photoresist at 2250 rpm, annealing at 115 ${}^{\circ }$C for 30 s, and exposing under a ultraviolet (UV) lamp with a single mask. After photoresist development with MF319, the substrate was annealed at 110 ${}^{\circ }$C for 150 s and the plasma lead features were etched with Cr etchant. The design allows for a 1 mm separation between the anode and cathode. The alumina ceramic material was chosen as the substrate for its ability to be cast into low-cost micro-structures, mechanical robustness, and high-voltage insulation.

## 3. Experimental Results

**Figure 2:**
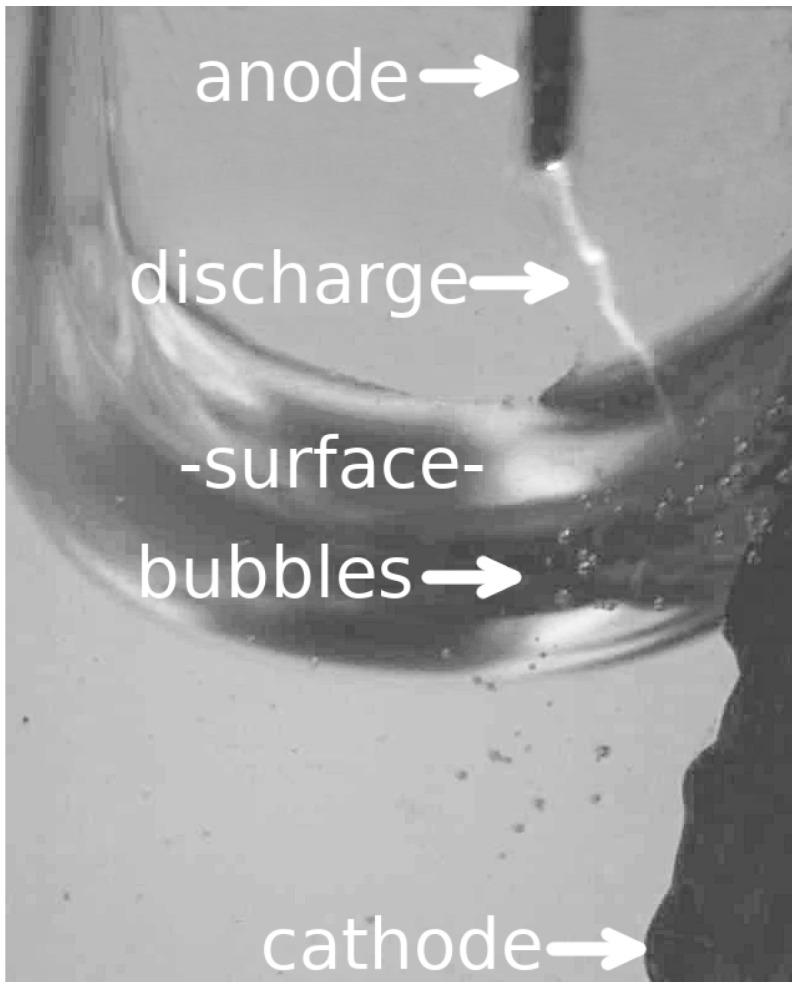
Response at the surface of the sample from a 1 mm microdischarge.

Concentrations of NaCl, CuCl, Ca(NO${}_{3}$)${}_{2}$, and CsOH were mixed in deionized H${}_{2}$O for testing the device. A variety of configurations were used in testing. For the data displayed, the configuration description follows. Spectroscopic analyses for all samples were performed in an air ambient at atmospheric pressure using 10 mL samples. Tungsten and copper were used as the anode and cathode, respectively. This accounts for the emission spectra that are observed in the figures that follow. An EMCO Q30N-5 DC to HV DC converter was used to amplify the voltage supplied to the device. With this particular device, an input of 5 V results in an output of 3000 V [12]. For all tests, the input to the DC to HV DC converter was set such that 3000 V was present on the tungsten anode. For each new sample, the anode and cathode material was replaced and the sample container was cleaned to eliminate residual responses. The anode was positioned approximately 1 mm above the sample’s surface. Activation of the microdischarge results in a formation of bubbles centered at the contact point and just under the surface. Figure 2 displays this. The “Handbook of Basic Atomic Spectroscopic Data” [13], *The Identification of Molecular Spectra* [14], and NIST’s online “Atomic Spectra Database” [15] were used to identify peaks.

**Figure 3:**
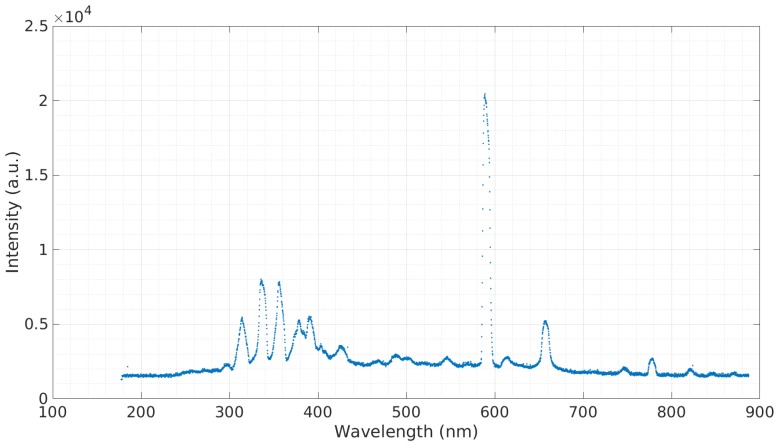
Emission spectra of 3.5% salinity simulated water.

**Figure 4:**
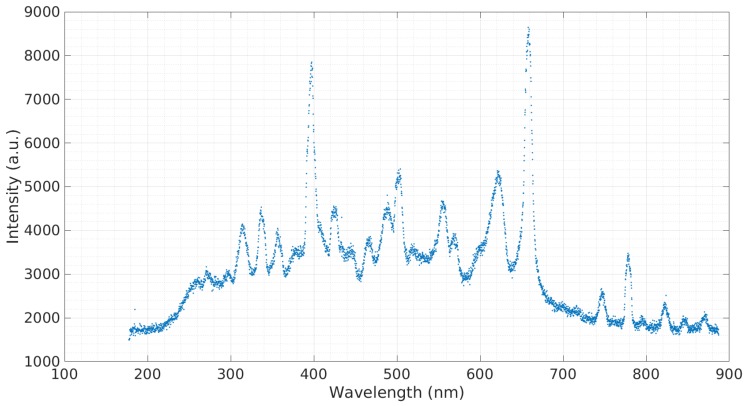
Emission spectra of Ca(NO${}_{3}$)${}_{2}$ in deionized water.

**Figure 5:**
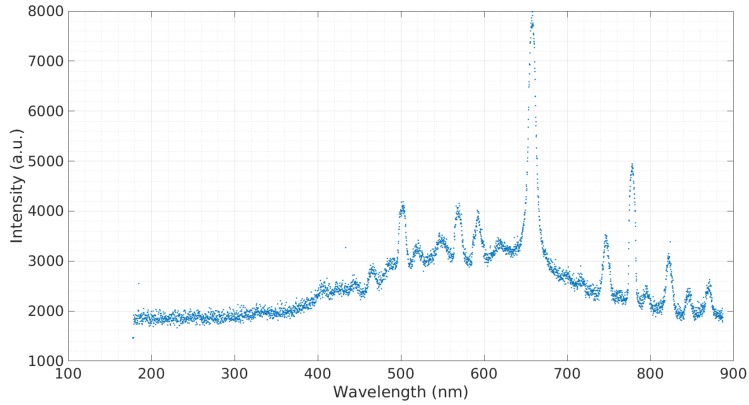
Emission spectra of CuCl in deionized water.

In order to obtain a baseline emission spectra, a sample of simulated seawater with 3.5% salinity was tested. Figure 3 shows this emission spectra. The prominent spectra observed at 590.3 nm belong to sodium (Na). A sample containing calcium was tested (Figure 4). The sample contained 3 g of Ca(NO${}_{3}$)${}_{2}$ to 20 g of deionized water. Calcium is a major ion in seawater, as it is critical for the formation of many structures, including skeletons and shells of corals and other organisms [16]. The most prominent calcium peak was observed at 396.60 nm. Additional calcium peaks were observed at 488.36 nm, 502.15 nm, and 518.65 nm. Copper is widely used in the construction of heat exchangers, pumps, valves, pipes, etc., and finds its way into seawater through corrosion [17]. A sample containing copper was tested (Figure 5). The sample contained 3 g of CuCl to 20 g of deionized water. The copper cathode was switched for an aluminum cathode so that the observed response would only be from the copper contained in the sample. Copper peaks at 429.26 nm and 794.43 nm were observed.

**Figure 6:**
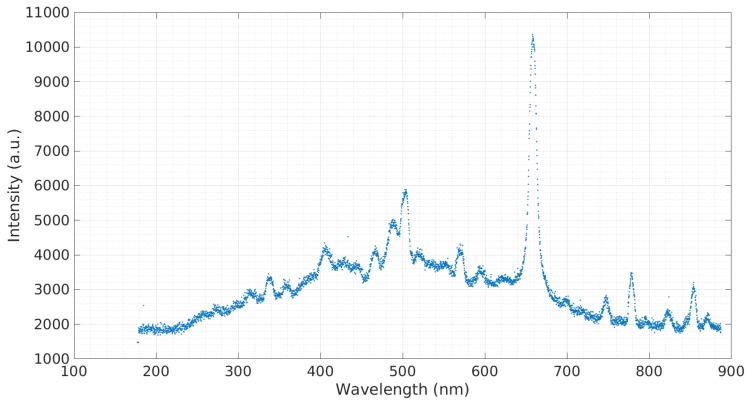
Emission spectra of the cesium-hydroxide sample.

**Figure 7:**
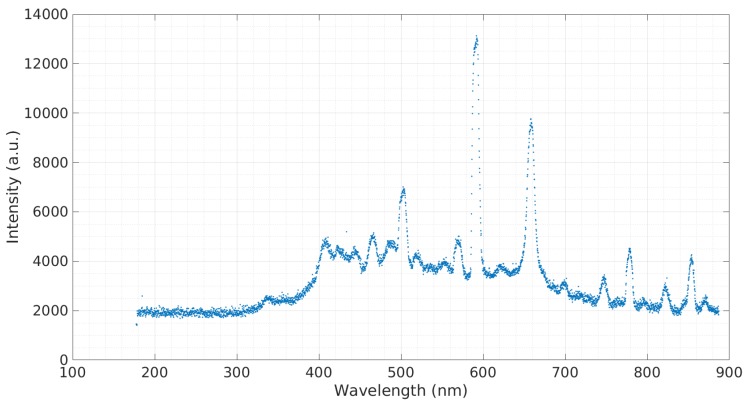
Cesium in saltwater.

A concentration of 3 mL CsOH to 10 mL deionized water was tested to obtain to the emission spectra seen in Figure 6. As seen in the graph, the most prominent cesium peak occurs at 853.92 nm with possible additional peaks occurring at 698.76 nm and 715.72 nm. To simulate cesium in saltwater, 0.3 g of NaCl was added to a 24.5 mL 310.3 ppm CsOH:H${}_{2}$O sample and tested, producing the emission spectra seen in Figure 7. Again, the cesium peaks at 853.9184 nm, 698.76 nm, and 715.72 nm are clearly observed, as well as the sodium peak at 590.36 nm.

**Figure 8:**
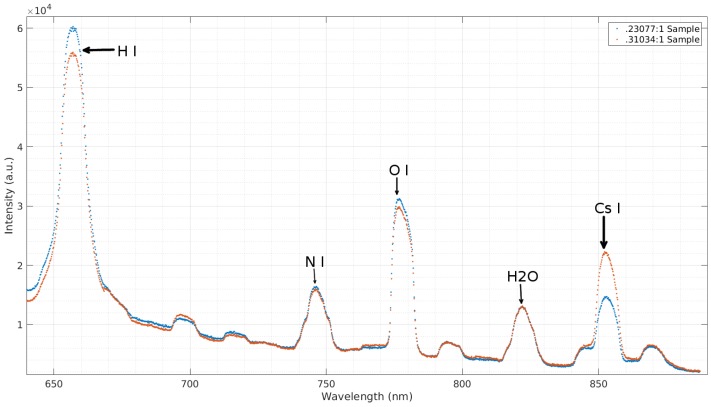
A comparison of two different concentrations of CsOH in deionized water.

In order to verify that cesium is in fact being observed and to show the ease at which the concentration of cesium can be determined relative to another sample, the emission spectra of samples with different CsOH to deionized water concentrations were measured. A comparison of spectra obtained from two samples with different concentrations can be seen in Figure 8. The two samples depicted contain 230.8 ppm and 310.3 ppm CsOH:H${}_{2}$O concentrations. A clear increase in the prominent Cs I peak at 853.92 nm can be observed, while other peaks, particularly those belonging to H${}_{2}$O and N I at 822.44 nm and 747.25 nm respectively, display no change. As expected, a slight increase in the H I and O I peaks at 658.37 nm and 778.03 nm is observed. This is due to the increase of these elements with the addition of CsOH.

**Figure 9:**
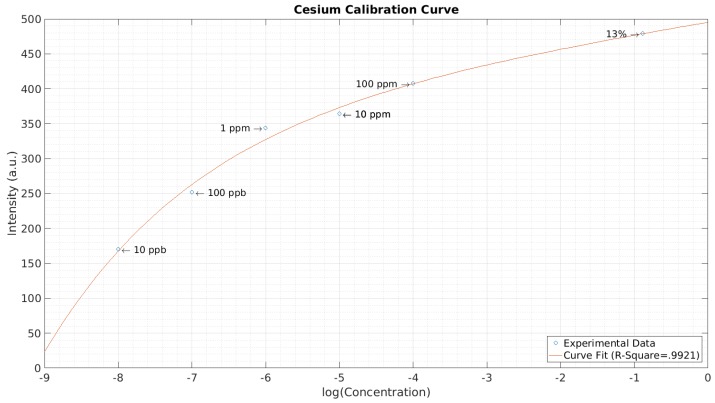
Calibration curve of cesium. The spectral peak at 853.9 nm was considered.

A calibration curve for cesium was also obtained and is depicted in Figure 9. Concentrations of 1 ppb, 10 ppb, 100 ppb, 1 ppm, 10 ppm, 100 ppm, and 13% were tested. After collecting the spectrum and adjusting to a baseline obtained when no power was supplied, the intensities of the 853.9 nm cesium peak were recorded. A curve fit performed in MatLab is also depicted in Figure 9. The experimental data collected follows an exponential trend. The cesium peak was not observable in the 1 ppb sample and is not included in the calibration curve. Therefore, a lower detection limit of 10 ppb was established.

## 4. Conclusions

The microdevice described in this article was shown to detect cesium contamination through observation of a spectral peak at 853.9 nm in samples with concentration as low as 10 ppb. Furthermore, the device allows for the differentiation of cesium to other elements, both naturally occurring and contaminants. By exciting a liquid sample with a high energy microdischarge and recording the spectral wavelengths emitted, the individual elements in the liquid become distinguishable. Through the matching of multiple peaks to known cesium spectra, the presence of cesium in a water sample is determined with certainty. Comparing the intensity of responses from samples provides a clear method in determining the concentration of cesium in relation to one another. Through this method, it is not possible to differentiate between cesium isotopes contained in the sample. However, this method provides a first-response style, on-site means for testing and monitoring water contamination. This device also provides a means for testing for the arrival of contamination propagating from the incident site in the days following the incident. Samples can later be shipped to laboratories for further testing. In the event of a disaster similar to the Fukushima collapse, this device, with its low cost of manufacturing and mobility, would be ideal for on-site detection of radioactive cesium contamination in seawater.
